# Extraction of Natural Antioxidants from the *Thelephora ganbajun* Mushroom by an Ultrasound-Assisted Extraction Technique and Evaluation of Antiproliferative Activity of the Extract against Human Cancer Cells

**DOI:** 10.3390/ijms17101664

**Published:** 2016-10-01

**Authors:** Dong-Ping Xu, Jie Zheng, Yue Zhou, Ya Li, Sha Li, Hua-Bin Li

**Affiliations:** 1 Guangdong Provincial Key Laboratory of Food, Nutrition and Health, School of Public Health, Sun Yat-sen University, Guangzhou 510080, China; xudp@mail2.sysu.edu.cn (D.-P.X.); zhengj37@mail2.sysu.edu.cn (J.Z.); zhouyue3@mail2.sysu.edu.cn (Y.Z.); liya28@mail2.sysu.edu.cn (Y.L.); 2 School of Chinese Medicine, The University of Hong Kong, Hong Kong 999077, China; u3003781@connect.hku.hk; 3 South China Sea Bioresource Exploitation and Utilization Collaborative Innovation Center, Sun Yat-sen University, Guangzhou 510006, China

**Keywords:** mushroom, *Thelephora ganbajun*, antioxidant, ultrasound-assisted extraction, response surface methodology, antiproliferative activity

## Abstract

The *Thelephora ganbajun* mushroom has been found to be a potential rich source of natural antioxidants. In this study, an ultrasound-assisted extraction (UAE) technique together with GRAS (generally recognized as safe) solvents (ethanol and water) was used to maximize the extraction of antioxidants from *Thelephora ganbajun*. Five extraction parameters (ethanol concentration, solvent to solid ratio, extraction time, temperature and ultrasound power) were investigated by single-factor experiments, and then a central composite rotatable design was employed to study interaction of three key extraction parameters. The optimum conditions were as follows: 57.38% ethanol, 70.15 mL/g solvent to solid ratio, 10.58 min extraction time, 40 °C extraction temperature and 500 W ultrasound power. Under the optimum conditions, the antioxidant activity obtained was 346.98 ± 12.19 µmol Trolox/g DW, in accordance with the predicted value of 344.67 µmol Trolox/g DW. Comparison of UAE with conventional maceration and Soxhlet extraction, the UAE method showed stronger extract efficiency in a shorter extraction time. These results showed that UAE was an effective technique to extract antioxidants from *Thelephora ganbajun*. Furthermore, the extracts obtained under the optimized conditions exhibited antiproliferative activities toward human lung (A549), breast (MCF-7), liver (HepG2) and colon (HT-29) cancer cells, especially for liver and lung cancer cells. In addition, rutin, 2-hydrocinnamic acid and epicatechin were identified in the extract, which might contribute to antioxidant and antiproliferative activities.

## 1. Introduction

Reactive oxygen species are the key components in the development of oxidative stress-related diseases. Natural antioxidants from foods, especially the polyphenolic forms, are the bioactive components that can quench reactive oxygen species [1,2]. Tumorigenesis is a multi-stage process [3]. These exogenous antioxidants can exert multiple effects at different stages of tumor progression, including blocking tumor cell transformation and proliferation and inducing tumor cell apoptosis by modulating multiple intracellular molecular targets [4,5]. In addition, radiation and chemical anticancer agents could exert serious side effects to patients. Antioxidant supplements as adjuvant therapy are consumed widely in order to prevent toxic side effects of cancer treatment [6]. Moreover, the consumption of antioxidant rich foods can also have beneficial impacts on other oxidative stress-related diseases, such as diabetes, cardiovascular diseases and ageing [1,2].

The mushroom *Thelephora ganbajun*, known as ‘‘Gan-Ba-Jun’’ in Chinese, is an ectomycorrhizal fungus that grows in symbiosis with pine trees in Yunnan Province of China [7,8,9]. It is a delicious and popular food for its unique flavor. Apart from its appreciated taste, *Thelephora ganbajun* is also attracting attention for its nutritional and medicinal properties, such as potent antioxidant, anti-insect, antibacterial and specific 5-lipoxygenase inhibitory activities [10,11,12]. In our previous study, *Thelephora ganbajun* was found to be a potential source of natural antioxidants for its strong antioxidant capacity among 49 edible macro-fungi from China [13]. Natural antioxidants in food could exhibit numerous bioactivities for human health and could also be used as additives in food industry. Thus, the effective extraction of natural antioxidants from *Thelephora ganbajun* is very important for its full utilization.

In order to improve the extraction efficiency, several extraction methods such as ultrasound-assisted extraction (UAE) [14], microwave-assisted extraction [15], enzyme-assisted extraction [16], and pressurized fluid extraction [17] have been developed to extract antioxidants from natural products. UAE is a green extraction technique due to its advantages over conventional methods, such as the higher extraction efficiency, the shorter extraction time and the lower solvent consumption [18,19,20]. The acoustic cavitation and mechanical effect produced by the ultrasound wave could enlarge the contact surface area between the material and solvent, and accelerate the penetration of solvent into the cell. Consequently, the bioactive components could rapidly diffuse from the material to the solvent [21]. The advantages of UAE have been shown in the extraction of antioxidants from *Stachys parviflora* [22], the polyphenols from chicory grounds [23], as well as the phenolics and the anthocyanins from blueberry (*Vaccinium ashei*) wine pomace [24].

Due to the physicochemical diversity of antioxidant components and the matrix effects, the antioxidants from different sources need different extraction approaches. The extraction yield of antioxidants varies widely according to the extraction conditions, such as concentration of solvent, solvent to solid ratio, ultrasound time, power and temperature [18,25]. Thus, the process parameters of UAE should be optimized. Generally, the extraction factors are screened firstly to find those parameters that have a significant impact on the extraction efficiency. Then, response surface methodology (RSM) should be conducted to maximize the extraction yield as a statistical multi-response optimization method. RSM is an effective statistic tool with minimized experimental trials and a 3D description of the interactions of parameters [18,26].

In this study, we focused our efforts on optimizing the ultrasound-assisted extraction parameters to obtain the extracts enriched with antioxidants using RSM with central composite rotatable design (CCRD). The comparison of UAE and two conventional extraction methods were performed to validate the extraction efficiencies of the optimal UAE. Furthermore, the antiproliferative activities of the extract toward the human lung (A549), breast (MCF-7), liver (HepG2) and colon (HT-29) cancer cells were also evaluated, which have not been reported in the literature.

## 2. Results and Discussion

### 2.1. Single Factor Experimental Analysis

#### 2.1.1. Influence of Ethanol Concentration

**Figure 1:**
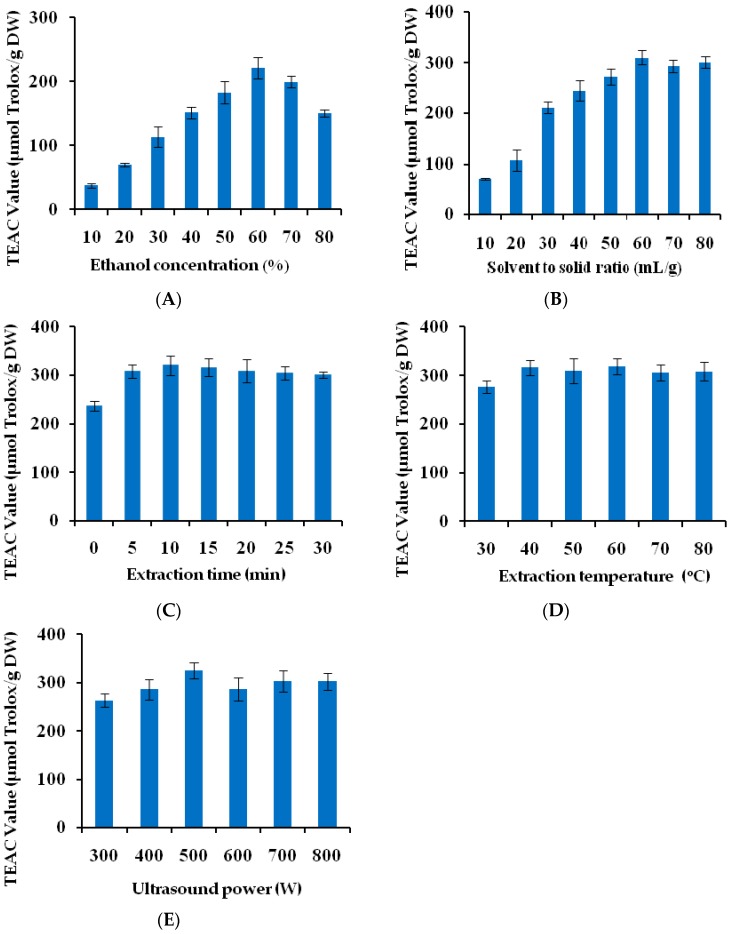
The effects of different process conditions on the Trolox equivalent antioxidant capacity (TEAC) values of *Thelephora ganbajun* extracts: (**A**) effect of ethanol concentration; (**B**) effect of solvent to solid ratio; (**C**) effect of extraction time; (**D**) effect of extraction temperature; and (**E**) effect of ultrasound power.

Ethanol aqueous was selected as the extraction solvent in this study because of its safety, easy accessibility and high affinity for antioxidants compared with other organic solvents [27,28,29]. Different ethanol concentrations had different polarities. Lower concentration of ethanol is suitable for extracting the polar antioxidant compounds, whereas higher concentration of ethanol favors the less polar ones [30]. The effects of ethanol concentration were determined in the range of 10%–80% (*v*/*v*) under the conditions of solvent to solid ratio 30:1 mL/g, ultrasound time 30 min, temperature 40 °C and power 800 W. As shown in Figure 1A, when the concentration of ethanol increased from 10% to 60%, the Trolox equivalent antioxidant capacity (TEAC) values increased significantly from 37.51 ± 3.62 to 221.29 ± 16.78 µmol Trolox/g DW (Trolox obtained from Sigma Chemical Co., St. Louis, MO, USA), while a decreased trend in the extraction efficiency of antioxidants was observed when the concentration of ethanol was higher than 60%. Thus, 60% ethanol was used in the subsequent experiment.

#### 2.1.2. Influence of Solvent to Solid Ratio

The effect of different solvent to solid ratio was determined in the range of 10–80 mL/g (*v*/*w*) under the conditions of ethanol concentration 60%, ultrasound time 30 min, temperature 40 °C and power 800 W. As shown in Figure 1B, the antioxidant activities increased significantly from 69.95 ± 1.80 to 310.55 ± 14.12 µmol Trolox/g DW as the ratio of solvent to solid increased from 10 to 60 mL/g. The results could be due to the fact that the higher ratio of solvent to solid dilutes the concentration of solutes in the extraction solution, increases the driving force between the solvent and cell, and then accelerates dissolution of more antioxidant compounds from the cell wall to the solvent [31]. However, as the ratio of solvent to solid was higher than 60 mL/g, reaching the saturation state, the value of TEAC almost did not vary remarkably. Therefore, 60 mL/g was selected as the optimal ratio of solvent to solid.

#### 2.1.3. Influence of Extraction Time

The effect of different extraction time (0, 5, 10, 15, 20, 25, 30 min) was investigated under the conditions of ethanol concentration 60%, solvent to solid ratio 60:1 mL/g, ultrasound temperature 40 °C and power 800 W. As seen from Figure 1C, when the extraction time changed from 0 min to 10 min, the TEAC values increased from 237.05 ± 9.68 to 320.36 ± 19.56 µmol Trolox/g DW. After 10 min, the extraction efficiency of antioxidants did not vary significantly and even decreased slightly at 30 min. A similar effect of ultrasonic time on the extraction of phenols from olive leaves was observed by Ahmad-Qasem et al. [32]. The results showed that within too short a time the antioxidants could not be completely extracted from *Thelephora ganbajun*, whereas the extraction time beyond 10 min was unnecessary, which could increase the energy costs and cause the degradation of the antioxidants [30,33]. Therefore, 10 min was adopted for the subsequent experiment.

#### 2.1.4. Influence of Temperature

Figure 1D shows the effect of different temperature (30, 40, 50, 60, 70 and 80 °C) on the extraction efficiencies under the conditions of ethanol concentration 60%, solvent to solid ratio 60:1 mL/g, ultrasound time 10 min and power 800 W. The antioxidant activities significantly enhanced when the temperature increased from 30 to 40 °C, and reached the peak (315.96 ± 15.54 µmol Trolox/g DW). However, when the temperature continued to rise, the TEAC values did not change significantly. The results indicated that the dissolution of the antioxidants from *Thelephora ganbajun* increased with the slight rise in extraction temperature, while the higher temperature (*T* > 40 °C) did not lead to the increase of antioxidant activities of *Thelephora ganbajun* extracts. Consequently, 40 °C was chosen as the optimal extraction temperature.

#### 2.1.5. Influence of Ultrasound Power

The effect of different ultrasound power (300, 400, 500, 600, 700 and 800 W) on the yield of antioxidants from *Thelephora ganbajun* was studied under the conditions of ethanol concentration 60%, solvent to solid ratio 60:1 mL/g, extraction time 10 min and temperature 40 °C. As shown in Figure 1E, with the ultrasound power increasing, the antioxidant activities started to increase, and reached the maximum (324.85 ± 16.72 µmol Trolox/g DW) at 500 W. Then, a slow decrease was followed by a further increase of the ultrasound power. This phenomenon could be explained by the fact that the higher ultrasound power would promote the mass transfer through producing stronger cavitation intensity of breaking the cell wall. However, the excessive power could cause degradation of the antioxidants in *Thelephora ganbajun* extracts [31]. Hence, 500 W was chosen as the optimal ultrasound power in the experiment.

Additionally, different batches of mushroom were tested, and similar results were obtained. Furthermore, antioxidant activity of the mushroom almost did not change within one month under the experimental conditions.

### 2.2. Response Surface Methodology Analysis

#### 2.2.1. Model Fitting

In the second part of this study, UAE parameters (ethanol concentration (X_1_), solvent to solid ratio (X_2_) and extraction time (X_3_)) were selected for the RSM optimization using CCRD design for the purpose of maximizing the extraction efficiency of antioxidants from *Thelephora ganbajun* and analyzing the combined effects of the three parameters. The central conditions chosen were 60% ethanol, 60 mL/g solvent to solid ratio and 10 min extraction time. The experiment design and the corresponding TEAC values under different UAE conditions are displayed in Table 1.

The experimental data of the CCRD were fitted to a quadratic equation by multiple regression analysis. The significance of each coefficient was evaluated through Student’s *t*-test (*p* < 0.05) and displayed in Table 2. For the three parameters, both the linear and quadratic effects of ethanol concentration (X_1_) were significant (*p* < 0.05) on the TEAC values, while extraction time (X_3_) only presented a significant quadratic effect (Table 3). The linear and quadratic terms of solvent to solid ratio (X_2_), as well as all of the interaction terms (X_1_X_2_, X_1_X_3_, X_2_X_3_,) did not show a significant impact (*p* > 0.05). Finally, a quadratic polynomial equation was attained (Equation (1)) where the non-significant variables were discarded (*p* > 0.05):

Y = −1318.02 + 48.29X_1_ − 0.39X_1_^2^ − 0.86X_3_^2^ (1)

The ANOVA in Table 2 revealed that the quadratic polynomial model generated was significant (*p* = 0.0035 < 0.05), and the lack of fit statistics was not significant (*p* = 0.5994 > 0.05), which indicated the variability of the model. The higher *R*^2^ value (closer to one) implies better correlation between the predicted value and the experimental results [34]. The value of *R*^2^ (0.86) in this model showed a good correlation between the experimental and predicted values. These results indicated that the fitted model was significant and adequate for the prediction of antioxidant activities of *Thelephora ganbajun* extracts within the tested ranges.

#### 2.2.2. Analysis of Response Surface Plots

**Figure 2:**
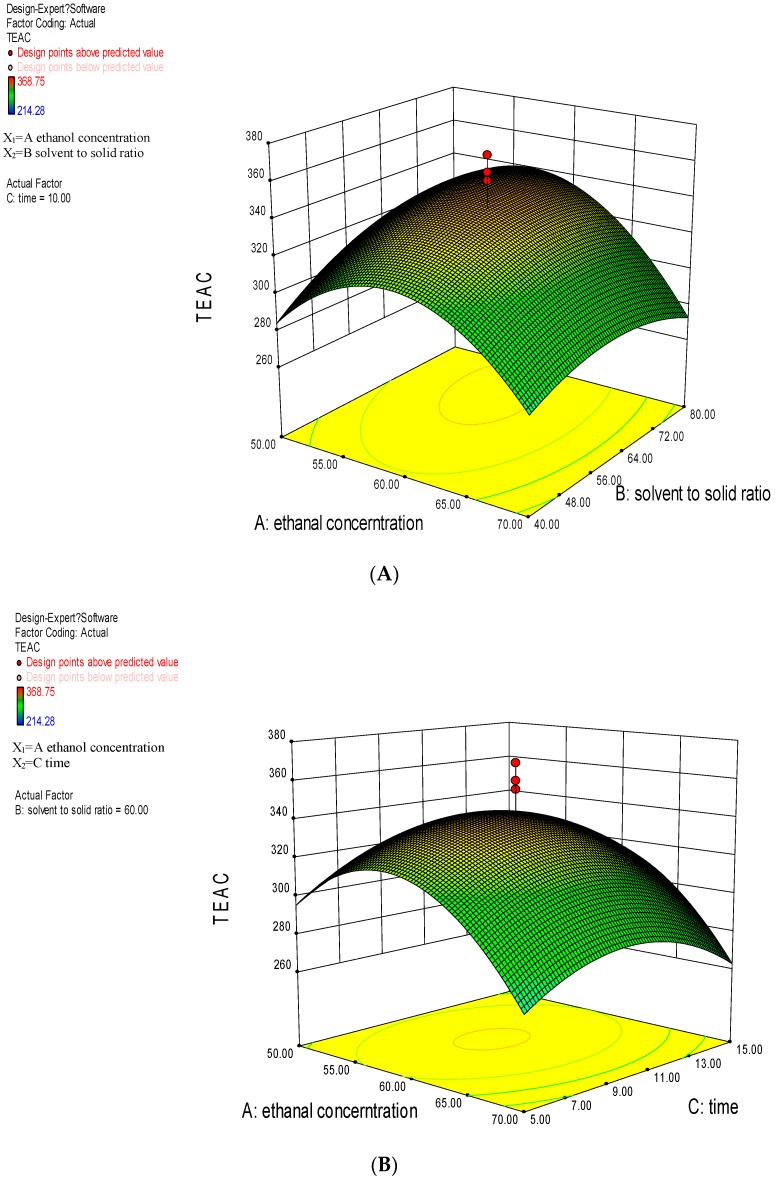

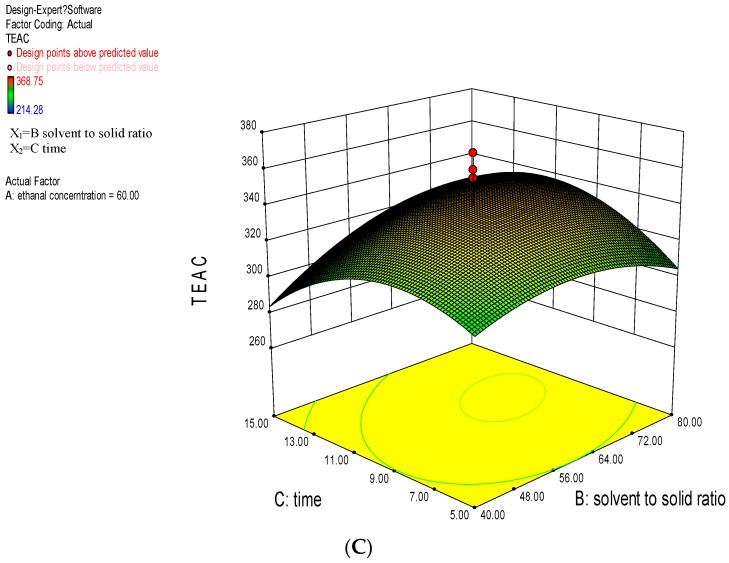
Response surface analysis for ultrasound-assisted extraction of antioxidants from *Thelephora ganbajun* with respect to ethanol concentration and solvent to solid ratio (**A**); ethanol concentration and extraction time (**B**); and solvent to solid ratio and extraction time (**C**).

The 3D response surface plots visually depicted the interactive influence of the two variables on the antioxidant activities of *Thelephora ganbajun* extracts, whereas the third one was fixed. The combined effect of ethanol concentration and solvent to solid ratio on extraction yield of antioxidant from *Thelephora ganbajun* is shown in Figure 2A. The response values were positive with the increase of solvent to solid ratio, while the response values increased at first and decreased afterwards with the increase of ethanol concentration. A marked quadratic effect of ethanol concentration was observed. The interactive influence of ethanol concentration and extraction time is presented in Figure 2B. At a fixed solvent to solid ratio, the increase of ethanol concentration and extraction time led to the initial increase of the response values and then decreased with their further increase. Figure 2C illustrates that ultrasonic time and ratio of solvent to solid had an impact on the response with a small positive slope at a fixed ethanol concentration, and a quadratic effect of extraction time was observed. The response surface plots (Figure 2) displayed were in accordance with ANOVA (Table 3). Based on the analysis of the surface plots, it could be clearly seen that the ethanol concentration was the dominant factor affecting the response, followed by the extraction time.

#### 2.2.3. Predicted Value Verification

In the fitted model, the maximum TEAC values of 344.67 μmol Trolox/g DW were predicted under the conditions: ethanol of 57.38%, solvent to solid ratio of 70.15 mL/g, extraction time of 10.58 min, extraction temperature of 40 °C and ultrasound power of 500 W. To further verify the accuracy of the model, the experimental rechecking was conducted six times under these optimal conditions, and the experimental TEAC value obtained was 346.98 ± 12.19 μmol Trolox/g DW. The results showed that the experimental results matched well with the predictive value. Thereby, the model obtained by RSM was effective and reliable for predicting the optimal antioxidant activities of *Thelephora ganbajun* extracts, and these optimal conditions obtained could be recommended to be applied to the extracting antioxidants from *Thelephora ganbajun*. In addition, antioxidant activity (346.98 ± 12.19 μmol Trolox/g) of the extract obtained under the optimized conditions was about nine times that obtained under un-optimized conditions (37.51 ± 3.62 μmol Trolox/g). Furthermore, the antioxidant activity was four times as much as that in the previous study (85.719 ± 1.932 μmol Trolox/g) [13]. Thus, this study markedly improved antioxidant activity of the extract, which allows more efficient utilization of *Thelephora ganbajun*.

#### 2.2.4. Comparison of Ultrasound-Assisted Extraction and the Conventional Extraction Methods

In order to estimate the extraction efficiency of ultrasound-assisted extraction for the antioxidants from *Thelephora ganbajun*, a comparative study was conducted between UAE and conventional maceration as well as Soxhlet extraction. From Table 3, it could be clearly seen that, compared with the conventional methods, UAE increased the antioxidant yield by more than 25% (204.34 ± 7.86, 276.76 ± 16.39 and 346.98 ± 12.19 μmol Trolox/g DW for maceration, Soxhlet and UAE, respectively), and greatly shortened the extraction time (24 h, 4 h and 10.58 min for maceration, Soxhlet and UAE, respectively). Furthermore, the total phenolic content (TPC) and total flavonoid content (TFC) of extract remarkably increased using UAE. The experimental data demonstrated that UAE was a more efficient method for extracting the antioxidants from *Thelephora ganbajun* with a higher extraction yield in a shorter extraction time. The strong antioxidant activity might be attributed to phenolic and flavonoid components existing in *Thelephora ganbajun* extract.

### 2.3. Antiproliferative Effects of Thelephora Ganbajun Extract

**Figure 3:**
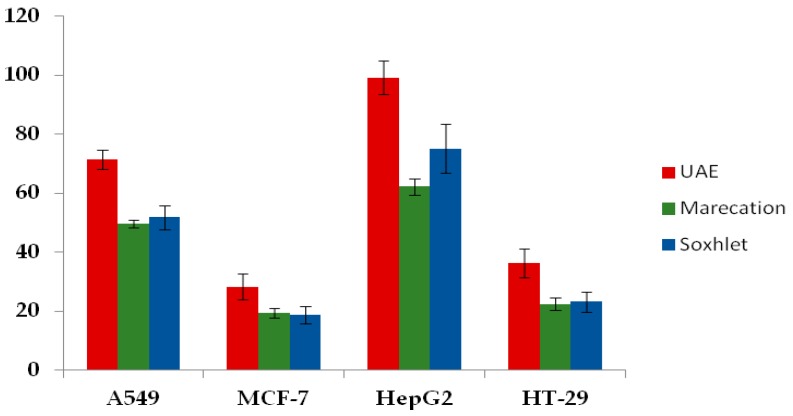
Antiproliferative capacities of *Thelephora ganbajun* extracts obtained by different extraction methods against four human cancer cell lines. UAE—Ultrasound-assisted extraction.

The antiproliferative activity toward cancer cells is generally regarded as an indicator of anticancer potential [4]. In this paper, *Thelephora ganbajun* were evaluated for their antiproliferative abilities against human lung (A549), breast (MCF-7), liver (HepG2) and colon (HT-29) cancer cell lines by an MTT (3-(4,5)-dimethylthiahiazo(-z-y1)-3,5-di-phenytetrazoliumromide) assay. The antiproliferative activities of *Thelephora ganbajun* extracts obtained by UAE, maceration and Soxhlet extraction methods were studied. As displayed from Figure 3, the results showed that among three extraction methods, UAE presented the strongest antiproliferative effects toward A549, MCF-7, HepG2 and HT-29 cancer cell lines, the inhibition percentage of the UAE extract against A549, MCF-7, HepG2 and HT-29 cancer cell lines was 71.4 ± 3.1, 28.3 ± 4.4, 99.1 ± 5.7 and 36.2 ± 4.9, respectively. Meanwhile, the four cancer cells exhibited different sensitivities to the antiproliferative activities of *Thelephora ganbajun* extract. Antioxidant bioactive components present in *Thelephora ganbajun* might be the main contributing factors for the difference [4]. A similar association between antioxidant activity and antiproliferative activities was also found in other studies. Olsson et al. noticed that the inhibition of HT29 and MCF-7 cancer cell proliferation in vitro correlated with levels of some antioxidant levels in fruit and berry extracts [35]. In MCF-7 cells, the anthocyanins might play a contributing role to the inhibition of proliferation. In another study, it was concluded that the antioxidant effects of polyphenols in the juice mixture played a contributing role in the inhibitory effects on the HepG2 cell [36].

### 2.4. Polyphenolic Compound Profile in Thelephora Ganbajun Extract

The polyphenols analysis could be useful in the bioactive studies involving *Thelephora ganbajun* mushroom. Characterization of polyphenols in the *Thelephora ganbajun* extract obtained under the optimized UAE conditions was carried out by HPLC-PDA with the standard compounds. Three antioxidant components were identified, including two phenolic compounds and one flavonoid (Table 4). The highest content of polyphenolic components detected was rutin (122.81 ± 5.23 mg/kg DW), followed by 2-hydrocinnamic acid (11.90 ± 1.02 mg/kg DW) and epicatechin (11.28 ± 1.56 mg/100 g DW). The high antioxidant activity of the extract might be attributed to the existence of these known and unknown polyphenolic components in the extract (Table 4). In another study, the presence of rutin has also been reported in Chanterelle (*Cantharellus cibarius*) mushroom [37].

## 3. Materials and Methods

### 3.1. Materials and Reagents

6-Hydroxy-2,5,7,8-tetramethylchromane-2-carboxylic acid (Trolox), 2, 2′-azinobis (3-ethylbenothiazoline-6-sulphonic acid) diammonium salt (ABTS), dimethylsulfoxide (DMSO), 3-(4,5-dimethylthiazole-2yl)-2,5-diphenyl tetrazolium bromide (MTT) and polyphenolic standards (epicatechin, chlorogenic acid, rutin, epicatechin gallate, coffeic acid, epigallocatechin, gallic acid, p-coumaric acid, resveratrol, ferulic acid, 2-hydrocinnamic acid, quercetin, kaempferol) were obtained from Sigma Chemical Co. (St. Louis, MO, USA). Dulbecco’s modified Eagle’s medium (DMEM, pH 7.4), fetal bovine serum and trypsin were obtained from Gibco (Invitrogen, New York, NY, USA). Human hepatoma (HepG2) and colon (HT-29) cancer cell lines were acquired from the No. 6 hospital affiliated with Sun Yat-Sen University (Guangzhou, China) and the School of Public Health, Sun Yat-Sen University, respectively. Human lung (A549) and breast (MCF-7) cancer cell lines were acquired from the No. 1 hospital affiliated to Sun Yat-Sen University. All other chemicals and solvents were of analytical grade.

*Thelephora ganbajun* was obtained from local markets in Yunnan Province, China, and was identified by Sha Li in School of Chinese Medicine, The University of Hong Kong (Hong Kong, China). The sample was dried at room temperature (residual moisture = 4.9%), ground into fine particles in an electric grinder (RHP-100; Ronghao Industry & Trade Co., Ltd., Yongkang, China) and sieved (0.15 mm particle size). The fine powder was stored at 4 °C under darkness until further use within one month.

### 3.2. Extraction of Antioxidants

#### 3.2.1. Ultrasound-Assisted Extraction

The extraction of antioxidants from *Thelephora ganbajun* was conducted in an ultrasonic bath device (Kj1012B; produced by Guangzhou Kejin Ultrasonic Instrument Factory, Guangzhou, China, bath frequency 28 kHz). The apparatus was equipped with a digital control system for irradiation power, time and temperature.

The powder of *Thelephora ganbajun* (0.1 g) was accurately weighed, and extracted with an appropriate amount of ethanol-aqueous solution. Then, the tubes with the mixture were immersed in a sonication water bath and were always fixed in the same position during sonication. Ultrasonic power (W), temperature (°C) and extraction time (min) were controlled from the panel of instrument. After the extraction, the mixtures were centrifuged at 4200× *g* for 30 min at 4 °C and stored under refrigeration (4 °C) for the antioxidant activity analysis within 48 h.

#### 3.2.2. Maceration

The maceration extraction proceeded according to the method described previously with minor changes [14]. After mixing with 57.38% ethanol (7.15 mL), the grounded sample (0.1 g) was extracted for 24 h in a shaking water bath at 25 °C and centrifuged at 4200× *g* for 30 min at 4 °C. The extracts obtained were stored at 4 °C prior to the antioxidant activity assay within 48 h.

#### 3.2.3. Soxhlet Extraction

The Soxhlet extraction proceeded according to the method described by Xu et al. with slight alteration [14]. The grounded sample (2 g) were extracted with 400 mL of 57.38% ethanol solution at 95 °C for 4 h, refluxing in a Soxhlet apparatus and centrifuged at 4200× *g* for 30 min at 4 °C. The extracts obtained were stored at 4 °C prior to the antioxidant activity assay within 48 h.

### 3.3. Experimental Design

#### 3.3.1. Single-Factor Experiments

To investigate the extraction efficiency of antioxidants from the *Thelephora ganbajun* mushroom under different ultrasound-assisted extraction conditions, single-factor tests were conducted to find the optimal conditions in the tested ranges, such as ethanol concentration (10%–80%), solvent/material ratio (10–80 mL/g), extraction time (0–30 min), extraction temperature (30–80 °C) and ultrasonic power (300–800 W).

#### 3.3.2. Response Surface Methodology

A five-level, three-variable central composite rotatable design (CCRD) was carried out to study the influence of three key extraction conditions: ethanol concentration (X_1_, %), solvent to solid ratio (X_2_, mL/g) and extraction time (X_3_, min) on the antioxidant activities of *Thelephora ganbajun* extracts. Ranges of three independent variables were selected on the basis of single factor experiment and their related codes and levels are listed in Table 5. The central composite rotatable design is present in Table 1. The design involves 20 experiments, which included six replicates in the central point. The data obtained was fitted to a quadratic polynomial equation by response surface regression and the equation is as follows:

Y = β_0_ + ∑β_i_X_i_ + ∑β_ii_X_i_^2^ + ∑β_ij_X_i_X_j_ (2)

where Y is the dependent variable (TEAC value); X_i_ and X_j_ are the independent variables; β_0_ is the interception; and β_i_, β_ij_ and β_ii_ are the linear, interactive and quadratic regression coefficients of the model, respectively. Analysis of variance (ANOVA) was conducted using Design Expert software (version 8.06.1, Stat-Ease, Minneapolis, MN, USA) to determine the statistical significance of the model. Three-dimensional surface plots were developed to study the interaction of three parameters while keeping a variable constant in the fitted model.

### 3.4. Measurement of Antioxidant Activity

The assay of ABTS radical-scavenging capacity was used to evaluate the antioxidant activity on the basis of the previous method [14]. At first, the ABTS^•+^ stock solution was prepared by mixing ABTS with potassium persulfate (*v*/*v* = 1:1), and incubating for 16 h under the darkness. Then, deionized water was added to the stock solution to make the absorbance be 0.70 ± 0.05 at 734 nm. For the spectrophotometric assay, 100 µL of the diluted sample was mixed with 3.8 mL of the ABTS^•+^ working solution, incubated for 6 min and determined the absorbance at 734 nm. The results of antioxidant activity were expressed as TEAC value and the unit of µmol Trolox/g dry weight (DW) was used.

### 3.5. Measurement of Total Phenolic Content

Total phenolic contents of *Thelephora ganbajun* extract were measured in accordance to the paper of Li et al. [38]. Briefly, 500 µL diluted sample was mixed with 2.5 mL diluted Folin–Ciocalteu solution (*v*/*v* =1:10) and the mixture was incubated for 4 min. Then, 2 mL of 75 g/L saturated Na_2_CO_3_ solution was added and the mixture was incubated for 2 h at room temperature. The absorbance of the reaction mixture was detected at λ_760 nm_. The reference standard used was gallic acid, and the total phenolic contents of extracts were expressed as mg GAE (gallic acid equivalent)/g of the *Thelephora ganbajun* sample.

### 3.6. Measurement of Total Flavonoid Content

Total flavonoid contents (TFC) of *Thelephora ganbajun* extract were determined in accordance with the literature of Kalia et al. [39]. In addition, 500 µL of sample extract was mixed with 1.5 mL of 95% ethanol (*v*/*v*), 0.1 mL AlCl_3_ (10%, *w*/*v*), 0.1 mL potassium acetate (1 M), and then 2.8 mL water was added to the mixture in a 5.0 mL volumetric flask. After 30 min, the absorbance of the reaction mixture was determined at λ_415 nm_. Quercetin was used as the reference standard, and the results were expressed as mg quercetin equivalent (mg QE)/g of *Thelephora ganbajun* sample.

### 3.7. Evaluation of Antiproliferative Abilities of Extracts toward Four Human Cancer Cells

#### 3.7.1. Sample Preparation

The grounded sample of *Thelephora ganbajun* (1 g) was extracted using the optimized UAE method above, and then the extract was evaporated to remove ethanol under vacuum using a rotary evaporator at 50 °C. The residual was dissolved with DMSO to 15 mL volume. Then, the extract was sterile filtered with a 0.22 µm millipore filter and stored at 4 °C before use.

#### 3.7.2. Cell Culture

A549, MCF-7, HepG2, and HT-29 cell lines were grown in DMEM (pH 7.4) containing 10% fetal bovine serum and 1% penicillin–streptomycin, and stored in a 5% CO_2_/37 °C incubator [40].

#### 3.7.3. Determination of Antiproliferative Effects

The antiproliferative abilities of the *Thelephora ganbajun* extract on four cancer cells were determined by MTT assay [40]. Cancer cells were seeded at a density of 1 × 10^5^ cells/mL in 96-well microtiter plates with 100 μL DMEM complete medium. After 24 h, 200 μL of 25 mg/mL sample extract was added to the culture medium and the culture medium was incubated for 48 h at 37 °C in a 5% CO_2_. Control wells contained the growth medium without sample, and blank wells received the growth medium without cancer cells. Then, the supernatant was discarded and the culture medium was washed with phosphate-buffered saline (PBS) twice, and 10 μL MTT solution (5 mg/mL) was added to each well. After 4 h, the untransformed MTT was removed and the formazan crystals were dissolved in 150 μL DMSO, and then shaken for 10 min on a table concentrator. The absorbance was determined at 490 nm by a microplate spectrophotometer (ELX800; BIOTEC, Wiesbaden, Germany). Antiproliferative effects were expressed as an inhibition percentage of a sample group in comparison with a control group.

### 3.8. High-Performance Liquid Chromatography with Photodiode Array Detection (HPLC-PAD) Analysis

Polyphenolic compounds in the extracts obtained by UAE were analyzed according to the method provided by Cai et al. [41] with slight changes. Separation of polyphenolic compounds was conducted on a Agilent Zorbax Extend-C18 column (250 × 4.6 mm, 5 μm) (Agilent, Santa Clara, CA, USA) and HPLC analysis was carried out using a Waters 1525 binary HPLC pump and a Waters 2996 photodiode array detector (Waters, Milford, MA, USA). The mobile phase consisted of solution A (0.1% formic acid-water solution) and solution B (methanol) with the gradient elution: 0–15 min, 95%–80% A, 15–20 min, 80%–70% A, 20–25 min, 70%–63% A, 25–40 min, 63%–60% A, 40–60 min, 60%–50% A. The operation was performed under the conditions of column temperature 40 °C, flow rate 0.8 mL/min, and UV detection at 200–600 nm. The polyphenols in the extracts were identified by comparing retention times and spectra with those of standard polyphenols. The contents of polyphenols in the extracts were determined by peak area under the maximum absorption wavelength. The results were expressed as mg/kg dry weight of *Thelephora ganbajun*.

### 3.9. Statistical Analysis

All the studies were conducted in triplicate and the results were recorded in the form of mean ± SD (standard deviation). Statistical analysis was carried out by Excel 2007 (Microsoft, Redmond, WA, USA) and Design Expert 8.06.1 (Stat-Ease, Minneapolis, MN, USA).

## 4. Conclusions

A green ultrasonic-assisted extraction technique for the extraction of natural antioxidants from *Thelephora ganbajun* has been developed. A central composite rotatable design was used in the optimization of the experimental parameters. In the present study, ethanol concentration of 57.38%, solvent to solid ratio of 70.15 mL/g, extraction time of 10.58 min, extraction temperature of 40 °C and ultrasonic power of 500 W were found to be optimal for extraction of antioxidants from *Thelephora ganbajun*. The maximum antioxidant activity of 346.98 ± 12.19 µmol Trolox/g DW was obtained under these optimal conditions. The good fit (*R*^2^ = 0.86) between the predicted and experimental values demonstrated that the response model was reliable to predict the optimal extraction conditions and the response value. Moreover, the UAE showed higher extraction efficiency in comparison with two conventional extraction techniques. These results obtained proved that the ultrasonic-assisted extraction method was an effective technique for extracting antioxidants from *Thelephora ganbajun*. Furthermore, it was found for the first time that the antioxidant rich extract from *Thelephora ganbajun* possessed significant antiproliferative activities toward human lung and liver cancer cells. In addition, rutin, 2-hydrocinnamic acid and epicatechin were identified in the extract via the HPLC analysis. These known and unknown polyphenolic components in the extract might contribute to antioxidant and antiproliferative activities.

## Figures and Tables

**Table 1:** Experimental designs and corresponding response values under different extraction conditions.

Run	X_1_ (Concentration of Ethanol, %)	X_2_ (Solvent to Solid Ratio, mL/g)	X_3_ (Ultrasound Time, min)	Y (TEAC Value, µmol Trolox/g DW)
1	70	40	5	263.08
2	50	40	5	259.96
3	70	40	15	214.3
4	50	80	5	312.87
5	60	60	10	314.54
6	60	26.36	10	307.88
7	43.18	60	10	247.7
8	60	60	1.59	279.67
9	60	60	10	336.34
10	50	40	15	255.81
11	70	80	5	236.27
12	76.82	60	10	214.28
13	60	60	10	355.12
14	70	80	15	273.64
15	60	60	10	368.75
16	60	60	10	307.28
17	50	80	15	312.87
18	60	60	18.41	281.12
19	60	93.64	10	300.01
20	60	60	10	359.59

**Table 2:** ANOVA of the fitted polynomial quadratic model.

Source	Sum of Squares	df	Mean Square	F Value	*p* Value	Significant
Model	33,516.29	9	3724.03	6.57	0.0035	significant
X_1_ (ethanol concentration)	3242.25	1	3242.25	5.72	0.0379	
X_2_ (solvent to solid ratio)	1223.51	1	1223.51	2.16	0.1727	
X_3_ (extraction time)	12.61	1	12.61	0.022	0.8845	
X_1_X_2_	749.62	1	749.62	1.32	0.2771	
X_1_X_3_	6.59	1	6.59	0.012	0.9163	
X_2_X_3_	1019.26	1	1019.26	1.8	0.2097	
X_1_^2^	21,871.78	1	21,871.78	38.56	0.0001	
X_2_^2^	2497.34	1	2497.34	4.40	0.0623	
X_3_^2^	6655.52	1	6655.52	11.73	0.0065	
Residual	5672.24	10	567.22			
Lack of fit	2501.52	5	500.30	0.79	0.5994	not significant
Pure error	3170.71	5	634.14			
Cor total	39,188.53	19				
R-squared				0.8553		
Adj R-squared				0.7250		

**Table 3:** The comparison of ultrasound-assisted extraction (UAE) with maceration and Soxhlet extraction.

Extracting Method	Ethanol Concerntration	Temperature	Time	TEAC Value (µmol Trolox/g DW)	TPC (mg GAE/g)	TFC (mg QE/g)
UAE	57.38%	40 °C	10.58 min	346.98 ± 12.19	91.51 ± 4.38	5.90 ± 0.27
Maceration extraction	57.38%	25 °C	24 h	204.34 ± 7.86	70.06 ± 3.63	3.84 ± 0.11
Soxhlet extraction	57.38%	95 °C	4 h	276.76 ± 16.39	72.68 ± 2.99	3.87 ± 0.12

**Table 4:** Polyphenolic components of *Thelephora ganbajun* flower extract.

Component	Retention Time (min)	Wavelength (nm)	Content (mg/kg DW)
Epicatechin	23.306	280	11.28 ± 1.56
Rutin	32.481	250	122.81 ± 5.23
2-Hydrocinnamic acid	35.410	280	11.90 ± 1.02
Unknown 1	15.855	260	9.14% *
Unknown 2	23.775	280	6.89% *
Unknown 3	38.166	320	9.72% *
Unknown 4	42.639	260	8.73% *

* represents relative peak area.

**Table 5:** Five levels of the independent variables.

Variable	Units	Symbol	Code Levels				
			−1.68	−1	0	1	1.68
Ethanol concentration	% (*v*/*v*)	X_1_	43.18	50	60	70	76.82
Solvent to solid ratio	mL/g	X_2_	26.36	40	60	80	93.64
Extraction time	min	X_3_	1.59	5	10	15	18.41
